# Efficacy of PET/CT in diagnosis of regional lymph node metastases in patients with colorectal cancer: retrospective cohort study

**DOI:** 10.1093/bjsopen/zrac090

**Published:** 2022-08-11

**Authors:** Ryohei Yukimoto, Mamoru Uemura, Takahiro Tsuboyama, Yuki Sekido, Tsuyoshi Hata, Takayuki Ogino, Norikatsu Miyoshi, Hidekazu Takahashi, Akira Kida, Mamoru Furuyashiki, Yuichiro Doki, Hidetoshi Eguchi

**Affiliations:** Department of Gastroenterological Surgery, Graduate School of Medicine, Osaka University, Osaka, Japan; Department of Gastroenterological Surgery, Graduate School of Medicine, Osaka University, Osaka, Japan; Department of Radiology, Graduate School of Medicine, Osaka University, Osaka, Japan; Department of Gastroenterological Surgery, Graduate School of Medicine, Osaka University, Osaka, Japan; Department of Gastroenterological Surgery, Graduate School of Medicine, Osaka University, Osaka, Japan; Department of Gastroenterological Surgery, Graduate School of Medicine, Osaka University, Osaka, Japan; Department of Gastroenterological Surgery, Graduate School of Medicine, Osaka University, Osaka, Japan; Department of Gastroenterological Surgery, Graduate School of Medicine, Osaka University, Osaka, Japan; Department of Radiology, Jinsenkai MI Clinic, Toyonaka, Osaka, Japan; Department of Radiology, Jinsenkai MI Clinic, Toyonaka, Osaka, Japan; Department of Gastroenterological Surgery, Graduate School of Medicine, Osaka University, Osaka, Japan; Department of Gastroenterological Surgery, Graduate School of Medicine, Osaka University, Osaka, Japan

## Abstract

**Background:**

Endoscopic and transanal local resection without lymph node dissection are treatment options for patients with a low risk of lymph node metastasis; however, some patients might have undiagnosed lymph node metastases before surgery. This retrospective study aimed to evaluate the efficacy of preoperative PET/CT for diagnosing regional lymph node metastasis.

**Methods:**

Patients who underwent curative resection with lymph node dissection for colorectal cancer at Osaka University between January 2012 and December 2015 were included. The cut-off values appropriate for diagnosing lymph node metastasis were calculated by way of a receiver operating characteristic (ROC) curves from maximum standard unit value (SUVmax) of main tumour, and lymph node short axis, and SUVmax of lymph node. The cut-off values of primary tumour SUVmax: 7, short-axis diameter of the lymph node at 7 mm, and lymph node SUVmax at 1.5 were set.

**Result:**

A total of 541 patients were included. Regional lymph node metastases were confirmed in resected specimens from 187 patients (35 per cent). With a primary tumour SUVmax of 7 used as a cut-off value, the sensitivity and specificity of regional lymph node metastasis were 70.1 per cent and 45.5 per cent respectively. With a cut-off short-axis diameter of the regional lymph node of 7 mm, the sensitivity and specificity of regional lymph node metastasis were 75.2 per cent and 82.6 per cent respectively, and with a cut-off regional lymph node SUVmax of 1.5, the sensitivity and specificity of regional lymph node metastasis were 78.6 per cent and 96.8 per cent respectively. When the diagnostic criteria were defined by a lymph node short-axis diameter of 7 mm or SUVmax of 1.5, the sensitivity and specificity were 87.4 per cent and 81.8 per cent respectively.

**Conclusion:**

Preoperative PET/CT is a useful modality for evaluating regional lymph node metastasis in patients with colorectal cancer.

## Teaser

This study aimed to evaluate the efficacy of PET/CT in the diagnosis of preoperative regional lymph node metastasis in patients with colorectal cancer. The results of this study highlight the high sensitivity and specificity of PET/CT for the preoperative diagnosis of regional lymph node metastasis, with a cut-off SUVmax of 1.5 being the optimal diagnostic value.

## Introduction

Colorectal cancer is the fourth most common cancer globally and second commonest cause for cancer death^1^. According to the WHO GLOBOCAN database, the number of patients with colorectal cancer in 2018 exceeded 1.8 million worldwide^2^. Accurate diagnosis of lymph node metastasis is critical for predicting prognosis and determining the treatment strategy for colorectal cancer^3,4^. In recent years, endoscopic and transanal local resection without lymph node dissection have been increasingly offered as part of a less-invasive treatment strategy with sphincter preservation^5,6^. In Japan, the 2019 Guidelines for Treatment of Colorectal Cancer state that local excision is indicated for cTis and cT1 cancer (slight invasion) located distal to the second Houston valve (peritoneal reflection). Histological assessment of resected specimens as part of a multidisciplinary discussion, guide the need for additional surgical resection when there is a possibility of lymph node metastasis^7^.

Lymph node metastases on postoperative pathological examinations were detected in approximately 7–15 per cent of patients with T1 colorectal cancer^8,9^, emphasizing the need for an accurate preoperative diagnosis.

Preoperative diagnosis of lymph node metastasis generally relies on the size, density, and morphology from CT/MRI examinations. Several reports discussing the accuracy of preoperative lymph node metastasis used short-axis diameters between 5 mm and 10 mm as cut-off values, but a consensus remains to be established^10–12^. CT is known to have limitations in the preoperative diagnosis of lymph node metastasis. A previous report stated that although preoperative CT accurately distinguishes between tumours confined to the bowel wall and those invading beyond the muscularis propria, it has significantly poorer performance for identifying nodal status^13^. Other predictors of lymph node metastasis include several pathological factors such as the depth of tumour invasion, lymphatic vessel invasion, and venous invasion; however, most of them are diagnosed after surgery using resected specimens and are difficult to apply in preoperative diagnosis^14^. A final decision about whether to perform regional lymph node dissection is made based on a comprehensive assessment of the curability and surgical risks.

^18^F-fluorodeoxyglucose (^18^F-FDG) PET/CT, which is a modality that reflects the biological activity of the tumour itself, has been used widely for preoperative evaluation of colorectal cancer^15,16^. A previous report demonstrated the efficacy of PET/CT for the preoperative diagnosis of lateral lymph node metastases in patients with rectal cancer^17^. This study aimed to assess the effectiveness of PET/CT and determine the optimal cut-off values for the preoperative diagnosis of regional lymph node metastasis in colorectal cancer.

## Methods

### Patients

This retrospective study included patients who underwent radical resection of colorectal cancer with lymph node dissection at Osaka University (Suita City, Japan) between January 2012 and December 2015. The exclusion criteria were no preoperative PET/CT, cancers with background colitis, localized recurrent tumour, local excision, and tumours histologically characterized as squamous cell or neuroendocrine carcinoma.

The study was approved by the Research Ethics Committee of Osaka University (approval ID 12418-6) and was performed following the Declaration of Helsinki and Good Clinical Practice Guidelines. All patients provided written informed consent before any form of clinical examination. This study was conducted in accordance with the equator network using Standards for Reporting of Diagnostic Accuracy Studies (STARD) 2015.

### PET/CT

PET imaging was performed briefly using ^18^F-FDG PET/CT by way of a Discovery 710 instrument (GE Health Japan). Three-dimensional data acquisition was initiated 60 min after the injection of 4.8 MBq/kg of FDG. The PET parameters included the maximal standardized uptake value (SUVmax). The SUVmax in the region of interest (ROI) was used as a representative value for assessing FDG uptake in the lesion^17^.

### Multidetector row CT

Multidetector row CT (MDCT) was sequentially performed after PET/CT on the same day. The MDCT parameters were as follows: tube voltage 120 kV, tube current 10–320 mA, using automatic exposure control in the *x*, *y*, and *z* planes with a noise index of 11.0, rotation speed of 0.6 s/r, helical pitch of 17.5 mm/r, and slice　thickness of 0.625 mm. The reconstruction intervals were set to 0.5 mm. For the contrast-enhanced MDCT images, a non-ionic contrast agent with an iodine concentration of 350 mg I/ml (Optiray, Guerbet Japan, Osaka, Japan) was infused at a flow rate of 4.0 ml/s followed by saline at the same rate during the arterial phase scanning with a dual-head injector (Stellant, Medrad, Indianola, Pennsylvania, USA). The volume of injected contrast agent was 100 ml for patients weighing less than 49 kg and 2.0 ml/kg for patients weighing 50 kg or more. To determine the arterial phase scan delay, a test injection with 10 ml contrast agent and 10 ml saline administered at the same rate was performed^18^.

### Evaluation of diagnostic performance

The diagnostic performance of the PET/CT SUVmax for regional lymph nodes in patients with colorectal cancer was examined. The regional lymph nodes were divided into three groups: the pericolic/perirectal, intermediate, and main lymph nodes. Evaluations were performed according to each region. In each group, the lymph node with the largest short axis on MDCT was defined as a lymph node with suspected metastasis, and the SUVmax was measured. The short axis of each lymph node was measured using Universal Viewer version 6.0 (GE Healthcare), and the SUVmax of the target lesion was measured using the ROI. The optimal cut-off value was determined using a receiver operating characteristic (ROC) curve. The cut-off value, which is located at the highest point on the vertical axis and the left end of the horizontal axis on the ROC curve, was calculated to maximize the sensitivity and specificity.

### Statistical analysis

The appropriate cut-off values for diagnosing metastasis were calculated using the ROC curves from the postoperative pathological examination, SUVmax obtained by PET/CT, and lymph node short-axis diameter from MDCT. The sensitivity, specificity, positive and negative predictive values, and diagnostic accuracy were calculated for each diagnostic criterion. A McNemar test of sensitivity and specificity was used to compare the detectability of each criterion. Logistic regression analysis was used to compare the usefulness between MDCT- and PET/CT-based diagnostic criteria described above for the preoperative diagnosis of lymph node metastasis. All statistical tests were performed using Pro 14 for Windows (SAS Institute, Cary, North Carolina, USA). Results were considered statistically significant at *P* < 0.05.

## Results

### Incidence of regional lymph node metastasis

Among the 664 patients, 123 were excluded due to no preoperative PET/CT (61 patients), cancer with background colitis (seven patients), localized recurrent tumour (21 patients), local excision (31 patients), and squamous cell or neuroendocrine carcinoma (three patients). A total of 541 patients and 1623 lymph nodes were examined (*Table 1*). Regional lymph node metastasis was identified on histopathological examination in 187 patients (35 per cent). The regional lymph node metastases to the pericolic/perirectal, intermediate, and main lymph nodes were detected in 181 (33.4 per cent), 43 (7.9 per cent), and 12 patients (2.2 per cent) respectively. There were 30 patients with metastases in two regions and 10 (1.8 per cent) with metastases in all three regions.

**Table 1:** Patient characteristics

Characteristics	*n* = 541
Age (years)*	67 (23–92)
Sex ratio (M:F)	314:58/227:42
**Tumour location**	
Caecum and ascending colon	131
Transverse colon	34
Descending colon	23
Sigmoid colon	143
Rectum	210
**Tumour differentiation**	
Well differentiated tubular	
adenocarcinoma	276
Moderately differentiated tubular	
adenocarcinoma	238
Poorly differentiated adenocarcinoma	16
Mucinous adenocarcinoma	11
**Preoperative therapy**	
Yes/no	46 (8.5)/495 (91.5)
**Pathological T category**	
T0/Tis/T1/T2/T3/T4	7/27/127/102/236/42
**Pathological N category**	
N0/N1/N2	354/128/59
**LN metastasis**	
Positive/negative	187 (35)/354 (75)
**Location of LN metastasis (positive/negative)**	
Pericolic/perirectal LN metastasis	181/360
Intermediate LN metastasis	43/498
Main LN metastasis	12/529
**Pathological stage (UICC TNM 8th)**	
0/I/II/III/IV	27/185/129/148/52
Primary tumour SUVmax mean (range)*	8.12 (4.62–12.29)
LN SUVmax mean (range)*	
Pericolic/perirectal LN metastasis	1.44 (0.36–13.46)
Intermediate LN metastasis	1.01 (0.37–8.45)
Main LN metastasis	0.84 (0.33–4.28)

*Values are *n* unless otherwise indicated. SUVmax, maximum standardized uptake value; LN, lymph node.

### ROC curve analysis for evaluation of cut-off values

**Fig. 1:**
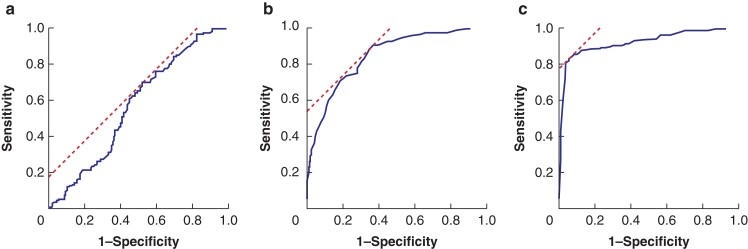
**a** Receiver operating characteristic (ROC) curve of the maximum standardized uptake value (SUVmax) in primary tumour as a predictor of pathological metastasis for regional lymph nodes. **b** ROC curve of the short axis diameter in the regional lymph nodes as a predictor of pathological metastasis for regional lymph nodes. **c** ROC curve of the SUVmax in the regional lymph nodes as a predictor of pathological metastasis for regional lymph nodes.

Among the 541 patients, the primary tumour SUVmax values were measured for 509 patients. Of the 541 patients, 32 who underwent additional resection after local excision were excluded because of the absence of preoperative PET/CT examination. These measurements were compared and evaluated against the postoperative histopathological examination. In the 541 patients, the lymph node with the largest short-axis diameter was extracted from each region (total of 1623 lymph nodes), and the optimal cut-off values of the primary tumour SUVmax, lymph node short-axis diameter, and lymph node SUVmax for the preoperative diagnosis of lymph node metastasis were determined using ROC analysis based on the postoperative pathological diagnosis results (*Fig. 1a–c*). The optimal cut-off values of the primary tumour SUVmax, lymph node short-axis diameter, and lymph node SUVmax were 7.0 mm, 7.0 mm, and 1.5 mm respectively. Based on the ROC analysis, the area under the curve values were 0.59 mm, 0.83 mm, and 0.91 mm respectively.

### Sensitivity, specificity, positive predictive value, negative predictive value, and accuracy

At a primary tumour SUVmax cut-off of 7.0, the sensitivity, specificity, positive predictive value, negative predictive value, and accuracy for lymph node metastasis diagnosis were 70.1 per cent, 45.5 per cent, 42.2 per cent, 72.9 per cent, and 54.4 per cent respectively (*Table 2*). At a lymph node with short-axis diameter cut-off of 7 on MDCT, the sensitivity, specificity, positive predictive value, negative predictive value, and accuracy for lymph node metastasis diagnosis were 75.2 per cent, 82.6 per cent, 42.6 per cent, 95.1 per cent, and 81.5 per cent respectively. At a 1.5 cut-off of lymph node SUVmax on PET/CT, the sensitivity, specificity, positive predictive value, negative predictive value, and accuracy of lymph node metastasis diagnosis were 78.6 per cent, 96.8 per cent, 80.6 per cent, 96.3 per cent, and 94.0 per cent respectively.

**Table 2:** Prediction of metastases using PET/CT based on histopathological diagnosis

	Histopathological diagnosis		
	Negative	Positive	Total
**PET/CT**			
Negative	148	55	203
Positive	177	129	306
**Total**	325	184	509

The cut-off of primary tumour SUVmax is set at 7. SUVmax, maximum standardized uptake value.

In patients with lymph node metastases, there was no significant difference in sensitivity between the short-axis diameters (cut-off value of 7 mm) and SUVmax value of lymph node (cut-off value of 1.5) by McNemar test (*P* = 0.198). Meanwhile, in patients without lymph node metastases, there was a significant difference in specificity between the short-axis diameters (cut-off value of 7 mm) and SUVmax of lymph node (cut-off value of 1.5) by McNemar test (*P* < 0.01). These results support that determining the lymph node SUVmax on PET/CT is useful for the preoperative metastasis diagnosis, and the cut-off value of 1.5 was appropriate for this purpose.

### Lymph node short axis on MDCT and SUVmax on PET/CT as composite criteria

**Fig. 2:**
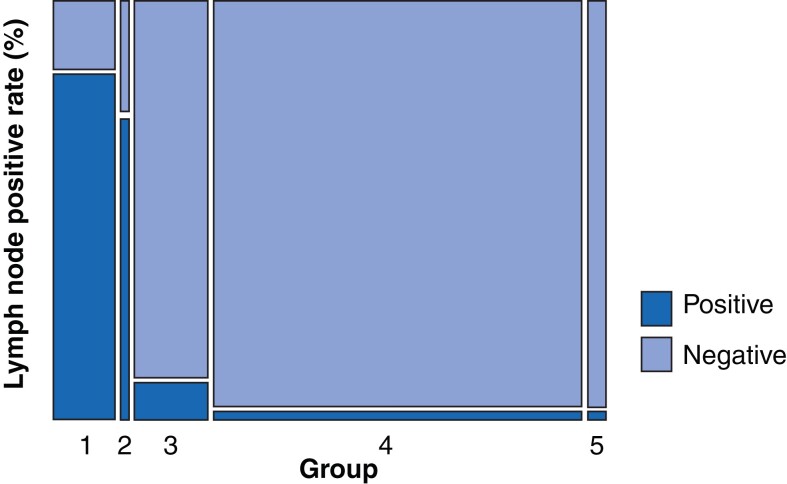
Distribution of patients in the five groups based on the lymph node short-axis diameter and SUVmax Proportion of patients positive for metastasis in the five groups.

A total of 1623 lymph nodes were classified into five groups using two criteria: 7 mm lymph node short axis on MDCT and 1.5 SUVmax on PET/CT. Group one included lymph nodes with a short axis of 7 mm or more and SUVmax of 1.5 or more (192 patients); group two, lymph nodes with a short axis less than 7 mm and SUVmax of 1.5 or more (40 patients); group three, lymph nodes with a short axis of 7 mm or more and SUVmax less than 1.5 (228 patients); group four, lymph nodes with a short axis less than 7 mm and SUVmax less than 1.5 (1079 patients); and group five, lymph nodes undetectable on MDCT (84 patients) (*Fig. 2*). The ORs for different groups were calculated with group four as reference. Compared with group four, the risk of lymph node metastasis was higher in group one (OR 168.26, 95 per cent c.i. 99.74 to 283.83) and group two (OR 95.45, 95 per cent c.i. 43.49 to 209.49). A comparison between lymph nodes with SUVmax of 1.5 or more or a short axis of 7 mm or more and those meeting neither of the criteria revealed that the risk of metastasis in the former group was higher than that in the latter group (OR 31.17, 95 per cent c.i. 20.76 to 46.80).

When the diagnostic criteria for metastasis was a lymph node SUVmax of 1.5 or more or short axis of 7 mm or more on MDCT and PET/CT, the sensitivity, specificity, positive predictive value, negative predictive value, and accuracy were 87.4 per cent, 81.8 per cent, 45.2 per cent, 97.4 per cent, and 82.7 per cent respectively.

## Discussion

Although endoscopic and transanal local resection without lymph node dissection have a role in the minimally invasive treatment of pT1 colorectal cancer, preoperative diagnosis of lymph node metastasis with higher accuracy is required. Recent reports have revealed that regional lymph node metastases on postoperative pathological examinations were found in approximately 7–15 per cent of patients with T1 cancer, and recurrence following local excision occurs in approximately 9 per cent of patients^8,9,19^. Colorectal cancer staging with PET/CT examinations has been reported to be effective and used widely in recent years. However, there is a paucity of reports on the effectiveness of PET/CT and optimal cut-off values for preoperative diagnosis of lymph node metastasis. The results of this study did not show the usefulness of the SUVmax value of the primary tumour for the preoperative diagnosis of lymph node status. This is in contrast with several reports indicating that the SUVmax of the primary tumour is useful for the diagnosis of lymph node metastasis and prediction of prognosis^20–22^.

The highest reliability was obtained when the short-axis diameter was used as a diagnostic criterion for lymph node metastasis. The results of the ROC analysis demonstrated that a short-axis diameter of 7 mm was the most appropriate criterion. A previous report showed that a cut-off value of 7 mm for lymph node short-axis diameter is a useful criterion for the diagnosis of lateral lymph node metastasis in patients with rectal cancer. Similarly, the present study showed that a 7 mm short-axis diameter was also an effective cut-off for the diagnosis of regional lymph node metastasis^17^. Several reports have described criteria of lymph node short-axis diameter for metastasis diagnosis, and the 7 mm cut-off used in this study is in keeping with these previous reports^7–9^. The usefulness of the lymph node SUVmax measurement for the diagnosis of lateral lymph node metastasis for patients with rectal cancer has been previously reported, with a lymph node SUVmax of 1.5 being the optimal cut-off value for this purpose^17^ as used in this study. These findings collectively suggest that a lymph node SUVmax of 1.5 is a universal criterion for diagnosing lymph node metastasis of colorectal cancer. The SUVmax criteria reported for lymph node metastasis are approximately 1.2–2.5, and the criterion of 1.5 is within this range as previously reported^23–26^.

Highly sensitive and reliable diagnostic criteria for lymph node metastasis are necessary to determine the appropriate treatment strategy in patients with colorectal cancer. The present study found that the combination of the two criteria with lymph node short-axis diameter on MDCT and the lymph node SUVmax on PET/CT, had high sensitivity for detecting lymph node metastasis. Among the 541 patients included, 157 (29 per cent) received no preoperative treatment and were found to have T0 or T1 tumour in postoperative pathological examinations. Among these patients, regional lymph node metastases were pathologically confirmed in 13 patients (8.2 per cent), and metastases were found in 14 lymph nodes among them. The lymph nodes from these patients were included in the analysis of the effectiveness of the established metastasis diagnostic criteria. In total, eight had a short-axis diameter of 7 mm or more on preoperative MDCT, and nine had a lymph node SUVmax of 1.5 or more on preoperative PET/CT. In summary, 11 of 14 lymph nodes met either of the two criteria.

This study has several limitations. There is no evidence of consistency between lymph nodes diagnosed as being positive for metastasis in postoperative pathological examinations and lymph nodes measured on MDCT and PET/CT. In this study, only one lymph node was targeted even in patients with metastases detected in multiple lymph nodes, and all metastatic lymph nodes could not be evaluated as target lymph nodes; however, this study examines the efficacy of PET/CT examinations for diagnosing regional lymph node metastasis and establishing the optimal cut-off values for this purpose in 500 or more colorectal cancer cases and 1500 or more regional lymph nodes with wider applicability to colonic cancers.

## Data Availability

The data sets generated and/or analysed during the present study are not publicly available due to the privacy of the enrolled participants, but these may be requested from the corresponding author upon reasonable request.

## References

[1] Bray F , Ferlay J, Soerjomataram I, Siegel RL, Torre LA, Jemal A. Global cancer statistics 2018: GLOBOCAN estimates of incidence and mortality worldwide for 36 cancers in 185 countries. CA Cancer J Clin 2018;68:394–424 30207593 10.3322/caac.21492

[2] International Agency for Research on Cancer. WHO Cancer Today. https://gco.iarc.fr/today/ (accessed 11 March 2019).

[3] Chang GJ , Rodriguez-Bigas MA, Skibber JM, Moyer VA. Lymph node evaluation and survival after curative resection of colon cancer: systematic review. J Natl Cancer Inst 2007;99:433–441 17374833 10.1093/jnci/djk092

[4] Schneider NI , Langner C. Prognostic stratification of colorectal cancer patients: current perspectives. Cancer Manag Res 2014;6:291–300 25061338 10.2147/CMAR.S38827 PMC4085313

[5] Sajid MS , Farag S, Leung P, Sains P, Miles WF, Baig MK. Systematic review and meta-analysis of published trials comparing the effectiveness of transanal endoscopic microsurgery and radical resection in the management of early rectal cancer. Colorectal Dis 2014;16:2–14 24330432 10.1111/codi.12474

[6] Marks J , Nassif G, Schoonyoung H, DeNittis A, Zeger E, Mohiuddin M et al Sphincter-sparing surgery for adenocarcinoma of the distal 3 cm of the true rectum: results after neoadjuvant therapy and minimally invasive radical surgery or local excision. Surg Endosc 2013;27:4469–4477 24057070 10.1007/s00464-013-3092-3

[7] Hashiguchi Y , Muro K, Saito Y, Ito Y, Ajioka Y, Hamaguchi T et al Japanese Society for Cancer of the Colon and Rectum (JSCCR) guidelines 2019 for the treatment of colorectal cancer. Int J Clin Oncol 2020;25:1–42 31203527 10.1007/s10147-019-01485-z PMC6946738

[8] Choi PW , Yu CS, Jang SJ, Jung SH, Kim HC, Kim JC. Risk factors for lymph node metastasis in submucosal invasive colorectal cancer. World J Surg 2008;32:2089–2094 18553050 10.1007/s00268-008-9628-3

[9] Macias-Garcia F , Celeiro-Muñoz C, Lesquereux-Martinez L, Gude-Sampedro F, Uribarri-Gonzalez L, Abdulkader I et al A clinical model for predicting lymph node metastasis in submucosal invasive (T1) colorectal cancer. Int J Colorectal Dis 2015;30:761–768 25700808 10.1007/s00384-015-2164-3

[10] Akasu T , Iinuma G, Takawa M, Yamamoto S, Muramatsu Y, Moriyama N. Accuracy of high-resolution magnetic resonance imaging in preoperative staging of rectal cancer. Ann Surg Oncol 2009;16:2787–2794 19618244 10.1245/s10434-009-0613-3

[11] Matsuoka H , Nakamura A, Sugiyama M, Hachiya J, Atomi Y, Masaki T. MRI Diagnosis of mesorectal lymph node metastasis in patients with rectal carcinoma. What is the optimal criterion? Anticancer Res 2004;24:4097–4101 15736458

[12] Ogura A , Konishi T, Cunningham C, Garcia-Aguilar J, Iversen H, Toda S et al Neoadjuvant (chemo)radiotherapy with total mesorectal excision only is not sufficient to prevent lateral local recurrence in enlarged nodes: results of the multicenter lateral node study of patients with low cT3/4 rectal cancer. J Clin Oncol 2019;37:33–43 30403572 10.1200/JCO.18.00032 PMC6366816

[13] Dighe S , Purkayastha S, Swift I, Tekkis PP, Darzi A, A’Hern R et al Diagnostic precision of CT in local staging of colon cancers: a meta-analysis. Clin Radiol 2010;65:708–719 20696298 10.1016/j.crad.2010.01.024

[14] Glasgow SC , Bleier JI, Burgart LJ, Finne CO, Lowry AC. Meta-analysis of histopathological features of primary colorectal cancers that predict lymph node metastases. J Gastrointest Surg 2012;16:1019–1028 22258880 10.1007/s11605-012-1827-4

[15] Watanabe A , Harimoto N, Yokobori T, Araki K, Kubo N, Igarashi T et al FDG-PET reflects tumour viability on SUV in colorectal cancer liver metastasis. Int J Clin Oncol 2020;25:322–329 31612350 10.1007/s10147-019-01557-0

[16] Sanli Y , Kuyumcu S, Ozkan ZG, Kilic L, Balik E, Turkmen C et al The utility of FDG-PET/CT as an effective tool for detecting recurrent colorectal cancer regardless of serum CEA levels. Ann Nucl Med 2012;26:551–558 22644560 10.1007/s12149-012-0609-0

[17] Yukimoto R , Uemura M, Tsuboyama T, Hata T, Fujino S, Ogino T et al Efficacy of positron emission tomography in diagnosis of lateral lymph node metastases in patients with rectal cancer: a retrospective study. BMC Cancer 2021;21:520 33962569 10.1186/s12885-021-08278-6 PMC8105987

[18] Ogino T , Takemasa I, Horitsugi G, Furuyashiki M, Ohta K, Uemura M et al Preoperative evaluation of venous anatomy in laparoscopic complete mesocolic excision for right colon cancer. Ann Surg Oncol 2014;21(Suppl 3):S429–S435. 24633663 10.1245/s10434-014-3572-2

[19] Antonelli G , Vanella G, Orlando D, Angeletti S, Di Giulio E. Recurrence and cancer-specific mortality after endoscopic resection of low- and high-risk pT1 colorectal cancers: a meta-analysis. Gastrointest Endosc 2019;90:559–569 31175875 10.1016/j.gie.2019.05.045

[20] Sasaki K , Kawasaki H, Sato M, Koyama K, Yoshimi F, Nagai H. Impact of fluorine-18 2-fluoro-2-deoxy-D-glucose uptake on preoperative positron emission tomography/computed tomography in the lymph nodes of patients with primary colorectal cancer. Dig Surg 2017;34:60–67 27454870 10.1159/000448222

[21] Ogawa S , Itabashi M, Kondo C, Momose M, Sakai S, Kameoka S. Prognostic value of total lesion glycolysis measured by 18F-FDG-PET/CT in patients with colorectal cancer. Anticancer Res 2015;35:3495–3500 26026116

[22] Shi D , Cai G, Peng J, Li D, Li X, Xu Y et al The preoperative SUVmax for (18)F-FDG uptake predicts survival in patients with colorectal cancer. BMC Cancer 2015;15:991 26689966 10.1186/s12885-015-1991-5 PMC4687154

[23] Bae SU , Won KS, Song BI, Jeong WK, Baek SK, Kim HW. Accuracy of F-18 FDG PET/CT with optimal cut-offs of maximum standardized uptake value according to size for diagnosis of regional lymph node metastasis in patients with rectal cancer. Cancer Imaging 2018;18:32 30217167 10.1186/s40644-018-0165-5 PMC6137872

[24] Tsunoda Y , Ito M, Fujii H, Kuwano H, Saito N. Preoperative diagnosis of lymph node metastases of colorectal cancer by FDG-PET/CT. Jpn J Clin Oncol 2008;38:347–353 18424814 10.1093/jjco/hyn032

[25] Tateishi U , Maeda T, Morimoto T, Miyake M, Arai Y, Kim EE. Non-enhanced CT versus contrast-enhanced CT in integrated PET/CT studies for nodal staging of rectal cancer. Eur J Nucl Med Mol Imaging 2007;34:1627–1634 17530248 10.1007/s00259-007-0455-9

[26] Yu L , Tian M, Gao X, Wang D, Qin Y, Geng J. The method and efficacy of 18F-fluorodeoxyglucose positron emission tomography/computed tomography for diagnosing the lymphatic metastasis of colorectal carcinoma. Acad Radiol 2012;19:427–433 22265721 10.1016/j.acra.2011.12.007

